# Drawing Parallels Among Past Public Health Crises and COVID-19

**DOI:** 10.1017/dmp.2021.202

**Published:** 2021-06-18

**Authors:** George W. Contreras, Brigitte Burcescu, Tiffany Dang, Jeanette Freeman, Nathan Gilbreth, Juliet Jacobson, Keerthana Jayaseelan, David S. Markenson

**Affiliations:** 1 Center for Disaster Medicine, New York Medical College, Valhalla, NY, USA; 2 Institute of Public Health, New York Medical College, Valhalla, NY, USA; 3 School of Medicine, New York Medical College, Valhalla, NY, USA

**Keywords:** COVID-19, Pandemics, Public Health Practice, Disaster Medicine, Emergency Preparedness

## Abstract

In the early stages of the coronavirus disease 2019 (COVID-19) pandemic, there were shortages of personal protective equipment (PPE) and health-care personnel across severely affected regions. Along with a lack of testing, these shortages delayed surveillance, and possible containment of the virus. The pandemic also took unprecedented tolls on the mental health of many health-care workers who treated and witnessed the deaths of critically ill patients. To address these effects and prepare for a potential second wave, a literature review was performed on the response of health-care systems during the influenza pandemics of 1918, 1957, 2009, and the epidemics of Ebola, severe acute respiratory syndrome (SARS), and Middle East respiratory syndrome (MERS). We can use lessons identified to develop a competent and effective response to the current and future pandemics. The public must continue to engage in proper health mitigation strategies, including use of face coverings, physical distancing, and hand washing. The impact the pandemic has had on the mental health of frontline health-care workers cannot be disregarded as it is essential in ensuring effective patient care and mitigating psychological comorbidities. The lessons identified from past public health crises can help contain and limit morbidity and mortality with the ongoing COVID-19 pandemic.

On December 31, 2019, a novel coronavirus, severe acute respiratory syndrome coronavirus 2 (SARS-CoV-2), was reported to the World Health Organization (WHO). More than one year later, according to Johns Hopkins COVID-19 Resource Center, as of May 2021, the United States is leading the world in the number of cases and deaths.^1^ The emergence of the novel SARS-CoV-2 virus and the subsequent coronavirus disease 2019 (COVID-19) pandemic puts a spotlight on the management of previous public health emergencies.

Over the past 110 y, society has experienced the influenza pandemics of 1918, 1957, 2009, and the epidemics of Ebola, severe acute respiratory syndrome (SARS), and Middle East respiratory syndrome (MERS). In this literature review, observations of the impact and response to COVID-19 in the United States are compared with past public health emergencies. Each crisis was investigated in the areas of personal protective equipment (PPE), health-care personnel shortage, and mental health of health-care workers and the public. A better understanding of previous crises can help optimize the response of the health-care system to potential COVID-19 subsequent waves and future public health emergencies.

## Methods

For this literature review, Google Scholar and PubMed were used to search for primary research and review articles based on the following search terms: “COVID-19,” “SARS,” “MERS”, “epidemic,” “1918 pandemic,” “1957 Influenza,” “2009 H1N1 Influenza,” “Ebola,” “Personal Protective Equipment,” “Mask usage,” “Healthcare Worker Shortage,” “Medical Students,” “Mental Health.” Other resources, such as the WHO, Johns Hopkins Coronavirus Resource Center, the Centers for Disease Control and Prevention (CDC), and cataloged newspapers were used to find accurate information on these public health emergencies. Inclusion criteria included articles dating back to 1918 that reviewed and discussed the key terms previously listed. Exclusion criteria included articles published in languages other than English and did not discuss the key terms as listed; however, there was no exclusion for method of publication.

### PPE

PPE, such as gowns, gloves, N95 respirators, surgical masks, and face shields, are designed to protect the wearer from exposure to a pathogen and to reduce spread of infection. These forms of protection date back to the 17th century when physicians used waxed leather head gear, gloves, suits, and boots, along with glass goggles, fisherman under garbs, and masks filled with dried herbs.^2^

There were many barriers to containing infection during past public health emergencies. The 3 that were studied include ineffective PPE, shortages of PPE, and a lack of compliance by the general public.

#### Effectiveness of PPE

In 1918, as the influenza virus spread throughout the United States, researchers tested the efficacy of face masks made of buttercloth, to contain the virus in a novel way. Buttercloth is a breathable soft, cotton fabric that is woven in a tighter manner than gauze. Three layers of buttercloth were discovered to be sufficient to prevent respiratory organisms from spreading to a nearby agar plate, even if a patient was actively coughing. One of the first hospitals to implement use of these buttercloth masks was The Hospital of the Rockefeller Institute.^3^

In 2003, the SARS epidemic specifically highlighted the use of PPE in limiting mortality and protecting frontline workers so that a surge capacity could be maintained.^4^ In 1 case control study performed in hospitals in Hong Kong, it was discovered that the use of surgical masks and N95s by health-care personnel was the most effective method to prevent the spread of SARS.^5^

The effectiveness of PPE for health-care personnel was also demonstrated during the MERS epidemic. A study done between 2013 and 2015 in a tertiary care institution in Saudi Arabia found that basic infection control measures taken by its health-care workers, including use of indicated PPE, were effective in reducing MERS transmission.^6^ Of the total 180 health-care workers tested, there were no positive results, whereas the general public had 16 of 694 individuals test positive.^6^ There is overlap between MERS and COVID-19 as they are both caused by coronaviruses with similar modes of transmission. In the COVID-19 pandemic, it was estimated that solely wearing a cloth mask decreases the reproduction number by approximately 8.6%, and this benefit may be even greater for essential workers and vulnerable populations.^7^

Inadequate PPE during the 2014 Ebola epidemic left health-care workers extremely vulnerable. In West Africa, the mortality rate for health-care personnel was significantly higher than that of the general population. In Liberia, there was a mortality rate of approximately 8% among health-care personnel, compared with 0.11% within the general population.^8^ Such a high rate among health-care workers has been primarily attributed to ineffective PPE. During the epidemic, a study assessed standard PPE barrier efficacy (ie, gloves, gowns, goggles) by introducing participants to a nonpathogenic virus. It revealed that more than 70% of the time, there was evidence of contamination on undergarments and skin beneath the PPE, even after proper procedural removal of the PPE. This underscores the need for proper PPE to protect those at the frontlines from exposure and prevention of disease spread.^9^

### Shortages of PPE

As H1N1 spread in 2009, the CDC attempted to mitigate PPE shortage by releasing 25% of the supplies (39 million pieces of PPE) in the Strategic National Stockpile.^10^ Despite the emphasis on PPE during previous public health emergencies, there was still a PPE shortage at the beginning of the COVID-19 pandemic.^11^ To mitigate the shortage, many health-care personnel were forced to reuse some PPE, such as N95 respirators. A study done in New York City in April 2020 concluded that 92% of respondents who worked with COVID-19 patients reported mask reuse or extended use.^11^ The previous standard for these types of PPE was limited to 1-time use, since re-using these diminishes efficacy. The N95 respirator needs to form a complete seal over the mouth and nose to prohibit virus penetration, and structural damage can compromise its effectiveness.^12^

### Use of Masks Among the General Public

As shown by these previous health crises, PPE use has been a well-established method to limit disease spread among health-care workers since the 17th century.^2^ Its use among the public, however, is also crucial in curbing the spread of the disease.

To limit the spread of the 1918 influenza pandemic, the American Red Cross released detailed instructions on making face masks at home. It included recommendations for 6 layers of gauze or thin cheese cloth, hemmed and tapered edges, and daily sterilization.^13^ Mask factories began to open up including 1 in Cleveland, Ohio, making up to 1000 masks a day. Many women worked in these mask factories, while schoolteachers and students helped with production during their vacations.^13^ Newspapers in Minnesota published a guide on how to wear masks, reminding citizens to cover their nose and mouth.^14^ Masks were urged by public officials among many states including Colorado, California, and Indiana. It began as a plea by means of public newspapers to wear masks and progressed to police enforcing masks with a fine of $50 in some places, such as Los Angeles, CA.^15–17^ Similarly, mask use by the public proved beneficial in reducing disease spread during the SARS epidemic. A study done in Beijing, China, demonstrated the benefit of wearing masks in preventing transmission of the virus. People who wore masks in public had a 70% lower risk of being diagnosed with SARS compared with those who did not.^18^

Despite the notably decreased viral transmission rates associated with proper mask use across previous pandemics and epidemics, government guidelines during the 1957 pandemic did not emphasize the importance of wearing masks. Rather, a vastly different approach was taken to prevent disease spread. Effort went into supplying a vaccine, while schools stayed open, travel remained unrestricted, and the population remained un-masked.^19^

During the COVID-19 pandemic, PPE garnered attention when public health officials emphasized the use of masks and face coverings by the public to contain the virus. Due to shortages of masks, however, the CDC specifically recommended the use of cloth and other forms of face coverings to avoid depleting surgical masks and N95 respirators for health-care personnel.^20^ From April to May 2020, 15 states plus Washington DC signed a mask mandate and there was a decline in the daily case growth rate. COVID-19 infections dropped by 0.9% in the first 5 d after the mask mandate, 1.4% in days 6-10, 1.7% in days 11-15, and 2.0% in days 16-20 in the states that enforced the mask mandate.^21^ However, some states did not implement mask wearing early in the pandemic. In a study done across 200 countries, the 24 countries which implemented a mask mandate within 20 d of the onset of their respective outbreaks had an average COVID-19 related mortality rate of 4.7 per million by August 9. In countries which implemented a mask mandate within 30 d, there was an average COVID-19 related mortality rate of 26.6 per million, compared with the United States, where there was no national mandate early in the pandemic, resulting in a mortality rate of 502 per million.^22^

By reviewing the events that occurred during past public health emergencies, lessons can be learned to better protect our health-care personnel and the public and mitigate the spread of disease through the use of sufficient, effective, and compliance with usage of PPE.

## Health-Care Personnel Shortage

Health-care personnel shortage became a significant issue in the United States during the first quarter of 2020. Emergency departments saw a high volume of critically ill patients and the demand for intensive care units (ICUs) rose due to the need for mechanical ventilation for the most critically ill.^23^ As hospitals became overwhelmed, so did the capacity of the staff, resulting in a need for outside help.

The effort to replace existing staff or to provide surge staff for hospitals during past public health crises was not always made, which created a significant barrier to delivering high quality care. During the pandemic of 1957 in the United Kingdom, if nurses and doctors contracted influenza, the wards they served were closed. While this may have lessened the spread of the virus from provider to patient, it also limited patient access to appropriate health care.^24^

### Using Medical Students

The 1918 pandemic began after the United States became involved in World War I. Nine thousand nurses were sent overseas, and thousands were deployed to military bases.^25^ As a result, there was a widespread health-care personnel shortage, making the fight against the 1918 influenza more difficult. The need for health-care personnel led to the use of medical students. In a firsthand account, Dr. Isaac Starr, who was beginning his third year at University of Pennsylvania School of Medicine, recalled how third- and fourth-year medical students were called into action in September of 1918, after only receiving 1 lecture on influenza.^26^ Students assembled beds from storage and prepared makeshift hospitals. Fourth-year students became the interns, while third-year students acted as nurses. With only 2 trained nurses on each shift, retired physicians, priests, clergymen, and nuns assisted.^27^

Like the 1918 influenza, the health-care personnel shortage during the COVID-19 pandemic led to the recruitment of medical students. In short-staffed areas, local medical schools instituted their own policies to increase personnel on the frontlines. For example, fourth-year medical students from New York University, Oregon Health and Sciences University, University of Massachusetts, Tufts University, Boston University, and New York Medical College graduated early to help with hospital staffing.^28–32^

Despite historical recruitment of medical students as health-care personnel during shortages, there is much disagreement regarding the clinical roles of medical students and the extent of their participation across varying institutions in routine medical care.^33^ In an effort to advocate for students, the American Association of Medical Colleges established interim recommendations in March 2020 that stated “medical students are students, not employees” and “unless there is a critical health-care workforce (HCW) need locally, we strongly suggest that medical students not be involved in any direct patient care activities”.^34^ As seen from past pandemics and epidemics, however, using the aid of medical students can significantly ameliorate the heavy burden on health-care personnel.

### Expanding the Scope of Practice

While the 1918 pandemic mitigated health-care personnel shortage by expanding the role of medical students, the 2009 pandemic extended the scope of practice of active health-care personnel.^35^ In November 2009, the Maryland governor issued an order allowing emergency medical technicians (EMT)-Paramedics and Cardiac Rescue Technicians to vaccinate patients.^35^

During the COVID-19 pandemic, response to the health-care personnel shortage was seen at both the federal and local institutional levels. Early in the pandemic, the US Department of Health and Human Services eased restrictions to allow licensed health-care providers to practice in another state in which they did not have a license.^36^ This early action facilitated an influx of physicians, nurses, and other health-care personnel to New York and New Jersey to assist within various departments in hospitals.^37^ In addition, during the COVID-19 pandemic, New York State allowed EMTs, licensed practical nurses, pharmacists, podiatrists, midwives, dentists, and dental hygienists to administer vaccinations against COVID-19.^38^ These changes in regulations call attention to ways that health-care workers expanded scope of practice can be used to supplement the shortage in specific care areas in future pandemics.

To further accommodate for the overflow of patients in hospitals and the shortage of health-care staff, the federal government deployed the hospital ship, United States Naval Ship (USNS) Comfort, and converted the Javits Center, a large convention center in New York City, into a makeshift hospital with military health-care personnel.^39^ In mid-July 2020, several states including Arizona, Texas, California, and Florida experienced persistent surges in cases and hospitals were overwhelmed and understaffed at many health-care facilities.^40^ As the cases in New York leveled off, some hospitals in New York sent health-care personnel to severely affected states such as Utah in August 2020.^41^ Throughout the pandemic, the military also sent resources to many states that needed support and resources.^42^

Previous public health emergencies caused shortages in health-care personnel that were similar to those seen during the COVID-19 pandemic. In planning for future public health emergencies, health-care systems may consider mirroring the mitigation strategies seen throughout history by using medical students, easing state licenses, expanding the scope of already practicing health-care personnel, using military assets, and shifting health-care providers from 1 region to another.

## Mental Health

The COVID-19 pandemic has underscored another facet of the health-care system: mental health. According to the CDC, as of June 2020, 40.9% of US adults were suffering from at least 1 adverse mental or behavioral health condition as result of the pandemic.^43^ Some of the most vulnerable groups in the population included women, elderly, individuals with premorbid psychopathology, and those with poor social support and/or low socioeconomic status.^44^ Another study showed that an individual’s mental health can alter health outcomes, when they are exposed to infections. It was found that patients with a higher positive attitude score had significantly lower infectious inoculations when exposed to influenza virus intranasally.^45^ The discussion of mental health in relation to public health emergencies is not only a pertinent matter but may also open other avenues in which the health-care system can improve its response to the current pandemic.

### General Public

The daily lives of the general population were severely altered by the pandemic of COVID-19. The rates of depression, suicide, anxiety, and other psychological comorbidities significantly increased during this period.^43^ During the COVID-19 outbreak, many people were forced to work from home, while millions lost their jobs and continue to be laid off. During the pandemic, the US unemployment reached a peak rate of 14.8% in April 2020, gradually decreasing to 6.7% by December 2020. With schools closed, families had to simultaneously provide childcare while working.^46^ In the last 2 wk of March 2020, there was a 60% increase in calls to the New York City branch of the National Alliance on Mental Illness, a nonprofit organization that provides support to those affected by mental illness. Other hotlines, including the Federal Disaster Distress Helpline saw unprecedented surges in calls, far exceeding any recent natural disasters.^47^

The profound impact of public health emergencies on the mental health of the public was noted during the 1918 and 1957 influenza pandemics. According to a Norwegian mental health researcher, Norwegian survivors of the 1918 influenza pandemic reported depression, distractibility, and difficulty coping at work.^48^

As localized outbreaks of the 1957 pandemic occurred in the United Kingdom, fear was so widespread throughout the population that the Minister of Health was asked to make a statement in an effort to allay fears. He declined twice, as he believed the pandemic was not actually spreading in the United Kingdom.^24^ This highlights a disconnect between the emotions and mental health of the population and the perception of infectivity and the impact that has by governing bodies.

Similarly, the MERS epidemic in 2015 sparked fear and anxiety in many people due to the uncertainty of treatment options and the absence of a vaccine. In a study of Korean citizens, it was found that the epidemic led to fears of daily activities, such as using public transportation and running errands. Of the 1000 Koreans surveyed who had participated in a 2-wk isolation, 80.2% reported fear of being infected and 46% reported emotional distress.^49^ These data show that isolation during an epidemic can have significant effects on emotional well-being.

Health-care systems have attempted to improve mental health in their own population by creating system wide changes to increase resources available to their own communities. During the 2009 pandemic, the National Biodefense Science Board (NBSB) identified major factors impacting the public’s mental health. They found that the population was most concerned about limited availability of vaccines and health care, conflicting news reports, increased US unemployment, and access to childcare. It also identified vulnerable populations including children, the elderly, the poor, and people with prior/current mental health and addictive disorders. To address these concerns across a wide population, the NBSB recommended the incorporation of mental health staff at clinics and vaccination centers to educate patients and to identify those experiencing significant distress.^50^

### Health-Care Personnel

During the COVID-19 pandemic, first responders and health-care personnel have faced the physical, mental, and emotional toll of treating a large number of critically ill persons, many of whom died in a short period of time. They witnessed the use of refrigerated trucks to store the bodies of those who had perished.^51^ At a time when the COVID-19 virus was not fully understood, health-care personnel were at the frontlines, without sufficient PPE. Individuals sacrificed going home to their families and slept in cars, garages, and hotels for weeks to months to protect their loved ones. The mental health impact of COVID-19 was seen in New York City in August 2020, with the suicides of 1 emergency physician, 2 paramedics, and 1 EMT.^52,53^

The adverse effects on mental health are not novel to the COVID-19 pandemic. High levels of comorbidities including posttraumatic stress disorder (PTSD), anxiety, and burnout were seen in those involved in direct patient care both during and after the SARS epidemic in 2003.^54,55^ There was a 28% increase in mental health comorbidities among physicians who provided care to patients with SARS compared with those who did not.^56^ During the 2014 Ebola epidemic, 1 in 4 health-care professionals involved in direct patient care experienced adverse mental health outcomes, including obsessive compulsions, depression, paranoid ideation, and interpersonal sensitivity.^54^

In addition to the hierarchical changes to improve mental health in the general population, similar changes are occurring to help health-care personnel. In memory of the emergency physician, Dr. Lorna Breen, lost to suicide in April 2020, the *Dr. Lorna Breen Health Care Provider Protection Act (Bill S.4349)* was introduced to the US Senate on July 29, 2020, with the goal of establishing training programs to encourage health-care personnel to seek help and support and to increase awareness of physician mental health and burnout.^53,57^

As of May 2020, the American Medical Association (AMA) released a set of 17 steps for an organization to take before, during, and after a crisis to reduce the psychosocial trauma of a crisis.^58^ The AMA recommends a systemic approach rather than an individual approach to mental health, emphasizing movement toward a focus on mental health on a larger, systemic scale. In accordance with such principles, health-care institutions across the United States began to increasingly focus on the mental health of health-care personnel. For example, in New York City, the Mount Sinai Health System organized a crisis support task force in March 2020 called the Center for Stress, Resilience, and Personal Growth, with the goal of promoting mental well-being of its staff.^58^ The task force focused on enhancing communication and developing strong psychosocial and mental health support systems. It offered services such as virtual psychologist-facilitated support groups; system-wide peer support hotline; psychiatry services; free apps for meditation, yoga, music therapy; and social networking groups.^58^ Similarly, Cody Regional Health of Wyoming established a designated “wellness area” featuring a meditation room, exercise room, and licensed therapists to support its health-care workers.^59^ In hopes of combatting the rising suicide rates, Atrium Health of North Carolina joined other hospitals, such as Cleveland Clinic, in establishing a Code Lavender program to provide rapid responses to emergent mental health needs. Code Lavender started to incorporate multiple therapeutic methods including music therapy, acupressure, and Reiki, as well as a wide range of staff from board certified chaplains to community volunteers.^59^

Evidence of mental health effects from past public health emergencies emphasizes the need to prevent, prepare, and address the impact of a crisis like COVID-19 at an institutional and individual level. While assuring individual access to mental health resources, an institutional approach is important, as traumatized health-care personnel may not recognize a decline in their mental health or know how to reach out for help. Creating a systemic culture of prioritizing mental health in the workplace brings awareness to these issues. Additionally, the feeling that 1 is valued by their supervisor has been found to reduce employee anxiety.^60^ This underscores the significant role that the health-care system plays in the mental health of health-care personnel. Systemic approaches toward mental health can provide data on how many workers are using provided resources and highlight the impact of the current pandemic on mental health with real time data. With an increased awareness of mental health in today’s society, there is the opportunity to be proactive and acknowledge the psychological impacts of the pandemic to help lessen the mental health burden.

## Limitations

There were certain limitations that were faced during the research process. While performing research during the ongoing pandemic, there were initially limited publications on COVID-19. This occasionally led to the use of reliable news sources as an alternative. In addition, limited peer reviewed primary literature was available for early influenza health crises. Because of this, other sources, such as archived local newspapers from various cities were used to gather information on the influenza of 1918. Finally, not every public health emergency identified in the abstract had valuable published information for each parameter covered in this study. For example, there was limited information on health-care worker shortage for the pandemic of 1957, and the Ebola, SARS, MERS epidemic, likely because they were not widespread enough to cause a significant worker shortage. These limitations should not be ignored and may be improved in future research on this topic, due to the constantly increasing database on COVID-19 related publications.

## Conclusion

There are several lessons that can be identified from past public health emergencies to decrease morbidity and mortality in the future. In this review, the influenza pandemics of 1918, 1957, and 2009 were analyzed along with the epidemics of Ebola, SARS, and MERS, specifically within the realms of shortages of PPE and health-care personnel, and the impact of mental health on health-care personnel and the public.

The influenza pandemics of 1918, 2009 H1N1, and SARS/MERS, Ebola epidemics have highlighted the need for sufficient and effective PPE. Shortage of effective PPE was a problem in the initial wave of COVID-19. To prevent this issue in future public health crises, proactive measures must be taken. This includes creating and maintaining national PPE stockpiles, recruiting private manufacturers to increase production during a crisis, and ensuring a clear plan for distribution.

Health-care worker shortages were previously seen in the influenza of 1918 and in the early phase of the COVID-19 pandemic. To address this issue, there needs to be an increase in the amount of health-care workers by bringing in military personnel and by temporarily relocating health-care workers to areas of greatest need. Furthermore, the scope of practice of current health-care workers must be broadened. This includes training medical students and other medical professionals to safely practice outside of their usual competencies.

Finally, past public health crises and the current COVID-19 pandemic have taken a toll on the mental health of both the general population and health-care personnel. In future public health emergencies, the effort to prioritize mental health and well-being should be proactive and not reactive. By encouraging system wide changes by means of the NBSB, AMA, and specific health-care systems, further declines in mental health of health-care workers can be prevented by increasing availability and accessibility to mental health resources. The lessons from past public health crises are crucial to optimizing the response to future obstacles that may arise during the COVID-19 pandemic. The outcomes outlined from these historical pandemics and epidemics can be used to better protect health-care workers and the public.

## References

[1] Johns Hopkins University of Medicine Coronavirus Resource Center. Website. COVID-19 Dashboard. https://coronavirus.jhu.edu/map.html. Accessed May 2, 2021.

[2] Oldfield E, Malwal SR. COVID-19 and other pandemics: how might they be prevented? ACS Infect Dis. 2020;6(7):1563-1566. doi: 10.1021/acsinfecdis.0c00291 32478500

[3] Doust B. Face masks in infections of the respiratory tract. The Journal of the American Medical Association. 1918;71(15):1216-1219. https://quod.lib.umich.edu/f/flu/7350flu.0016.537/--face-masks-in-infections-of-the-respiratory-tract?q1=mask&view=image&seq=1&size=100. Accessed April 22, 2021.

[4] Goldfrank L, Liverman C. Preparing for an Influenza Pandemic. Washington, DC: The National Academies Press; 2008.

[5] Seto WH, Tsang D, Yung R, et al. Effectiveness of precautions against droplets and contact in prevention of nosocomial transmission of severe acute respiratory syndrome (SARS). Lancet. 2003;361(9368):1519-1520. doi: 10.1016/S0140-6736(03)13168-6 12737864 PMC7112437

[6] Butt TS, Koutlakis-Barron I, AlJumaah S, et al. Infection control and prevention practices implemented to reduce transmission risk of middle east respiratory syndrome-coronavirus in a tertiary care institution in Saudi Arabia. Am J Infect Control. 2016;44(5):605-611. doi: 10.1016/j.ajic.2016.01.004 26922892 PMC7115292

[7] Worden L, Wannier R, Blumberg S, et al. Estimation of effects of contact tracing and mask adoption on COVID-19 transmission in San Francisco: a modeling study. medRxiv. 2020. doi: 10.1101/2020.06.09.20125831

[8] Evans DK, Goldstein M, Popova A. Health-care worker mortality and the legacy of the Ebola epidemic. Lancet Glob Health. 2015;3(8):e439-e440. doi: 10.1016/S2214-109X(15)00065-0 26163833

[9] Weber DJ, Fischer WA, Wohl DA, et al. Protecting healthcare personnel from acquiring Ebola virus disease. Infect Control Hosp Epidemiol. 2015;36(10):1229-1232. doi: 10.1017/ice.2015.205 26403694 PMC5656048

[10] IOM (Institute of Medicine). 2011. Preventing tranmission of pandemic influenza and other viral respiratory diseases: Personal protective equipment for healthcare personnel. Washington, DC: The National Academies Press. 24983058

[11] Breazzano MP, Shen J, Abdelhakim AH, et al. Resident physician exposure to novel coronavirus (2019-nCoV, SARS-CoV-2) within New York City during exponential phase of COVID-19 pandemic: report of the New York City residency program directors COVID-19 research group. *medRxiv*. 2020:2020.04.23.20074310. doi: 10.1101/2020.04.23.20074310

[12] Burgess A, Horii M. Risk, ritual and health responsibilisation: Japan’s ‘safety blanket’ of surgical face mask-wearing. Sociol Health Illn. 2012;34(8):1184-1198. doi: 10.1111/j.1467-9566.2012.01466.x 22443378

[13] Carter K. Behind scenes in a flu mask factory. The Cleveland Press. October 26, 1918:6. https://quod.lib.umich.edu/f/flu/0380flu.0002.830/1/--behind-scenes-in-a-flu-mask-factory?rgn=full+text;view=image;q1=mask. Accessed April 28, 2021.

[14] St. Paul Pioneer Press. How to wear mask and beat influenza. November 6, 1918:7. https://quod.lib.umich.edu/f/flu/9600flu.0010.069/1/--how-to-wear-mask-and-beat-influenza?page=root;rgn=full+text;size=100;view=pdf;q1=mask. Accessed April 21, 2021.

[15] Rocky Mountain News. Epidemic closing order revoked; masks urged to stop disease spread. November 24, 1918:1,4. https://quod.lib.umich.edu/f/flu/7090flu.0003.907/1/--epidemic-closing-order-revoked-masks-urged-to-stop-disease?rgn=full+text;view=image;q1=mask. Accessed April 18, 2021.

[16] The Denver Post. Police will enforce flu masking order. November 25, 1918:1-2. https://quod.lib.umich.edu/cgi/t/text/idx/f/flu/0210flu.0004.120/1/--police-will-enforce-flu-masking-order?rgn=subject;view=image;q1=Denver%2C+Colorado. Accessed April 18, 2021.

[17] Los Angeles Evening Herald. Big decline in ‘flu’ cases; mask law action held up. January 24, 1918:11. https://quod.lib.umich.edu/f/flu/7350flu.0005.537/1/--big-decline-in-flu-cases-mask-law-action-held-up?rgn=full+text;view=image;q1=mask. Accessed April 24, 2021.

[18] Wu J, Xu F, Zhou W, et al. Risk factors for SARS among persons without known contact with SARS patients, Beijing, China. Emerg Infect Dis. 2004;10(2):210-216. doi: 10.3201/eid1002.030730 15030685 PMC3322931

[19] Henderson DA, Courtney B, Inglesby TV, et al. Public health and medical responses to the 1957-58 influenza pandemic. Biosecur Bioterror. 2009;7(3):265-273. doi: 10.1089/bsp.2009.0729 19656012

[20] Fisher KA, Barile JP, Guerin RJ, et al. Factors associated with cloth face covering use among adults during the COVID-19 pandemic — United States, April and May 2020. MMWR Morb Mortal Wkly Rep. 2020;69:933-937. doi: 10.15585/mmwr.mm6928e3 32673303

[21] Lyu W, Wehby GL. Community use of face masks and COVID-19: evidence from a natural experiment of state mandates in the US. Health Aff (Millwood). 2020;39(8):1419-1425. doi: 10.1377/hlthaff.2020.00818 32543923

[22] Leffler CT, Ing E, Lykins JD, et al. Association of country-wide coronavirus mortality with demographics, testing, lockdowns, and public wearing of masks. Am J Trop Med Hyg. 2020;103(6):2400-2411. doi: 10.4269/ajtmh.20-1015 33124541 PMC7695060

[23] Baugh JJ, White BA, McEvoy D, et al. The cases not seen: patterns of emergency department visits and procedures in the era of COVID-19. *Am J Emerg Med.* 2020. doi: S0735-6757(20)30964-5 10.1016/j.ajem.2020.10.081 PMC7644188 33189517

[24] Jackson C. History lessons: The Asian flu pandemic. Br J Gen Pract. 2009;59(565):622-623. doi: 10.3399/bjgp09X453882 22751248 PMC2714797

[25] Keeling AW. “Alert to the necessities of the emergency”: U.S. nursing during the 1918 influenza pandemic. Public Health Rep. 2010;125 Suppl 3(Suppl 3):105-112. doi: 10.1177/00333549101250S313 PMC2862339 20568572

[26] Starr I. Influenza in 1918: recollections of the epidemic in Philadelphia. Ann Intern Med. 2006;145(2):138-140. doi: 10.7326/0003-4819-145-2-200607180-00132 16801626

[27] Johnson NP, Mueller J. Updating the accounts: Global mortality of the 1918-1920 “Spanish” influenza pandemic. Bull Hist Med. 2002;76(1):105-115. doi: S1086317602101050 11875246 10.1353/bhm.2002.0022

[28] NYMC News and Events. Members of the SOM class of 2020 graduate early and enter the fight against COVID-19. ˜https://www.nymc.edu/news-and-events/news-archives/members-of-the-som-class-of-2020-graduate-early-and-enter-the-fight-against-covid-19-.php#:˜:text=On%20April%208%2C%20a%20month,Class%20of%202020%20graduated%20early.&text=%22Since%20the%20COVID%2D19%20crisis,spirit%20of%20volunteerism%20and%20humanism. Accessed April 29, 2021.

[29] NYU Langone Health. Early graduation at NYU Grossman school of medicine sends new doctors to join COVID-19 fight. ˜https://nyulangone.org/news/early-graduation-nyu-grossman-school-medicine-sends-new-doctors-join-covid-19-fight#:˜:text=NYU%20Grossman%20School%20of%20Medicine%20was%20the%20first%20medical%20school,disease%20(COVID%2D19). Accessed April 30, 2021.

[30] Murphy B. COVID-19 and early medical school graduation: a primer for M4s. American Medical Association Web site. https://www.ama-assn.org/residents-students/residency/covid-19-and-early-medical-school-graduation-primer-m4s. Accessed April 30, 2021.

[31] Murphy B. COVID-19: states call on early medical school grads to bolster workforce. American Medical Association Web site. https://www.ama-assn.org/delivering-care/public-health/covid-19-states-call-early-medical-school-grads-bolster-workforce. Accessed April 28, 2021.

[32] O’Byrne L, Gavin B, McNicholas F. Medical students and COVID-19: the need for pandemic preparedness. J Med Ethics. 2020;46(9):623-626. 32493713 10.1136/medethics-2020-106353 PMC7316103

[33] Miller DG, Pierson L, Doernberg S. The role of medical students during the COVID-19 pandemic. Ann Intern Med. 2020;173(2):145-146. doi: 10.7326/M20-1281 32259194 PMC7151405

[34] Whelan A, Prescott J, Young G, et al. Interim guidance on medical students’ participation in direct patient contact activities: principles and guidelines. Association of American Medical Colleges. 2020. https://lcme.org/wp-content/uploads/filebase/March-30-2020-Interim-Guidance-on-Medical-Students-Participation-in-Direct-Patient-Contact-Activities.pdf. Accessed April 2, 2021.

[35] Courtney B, Morhard R, Bouri N, et al. Expanding practitioner scopes of practice during public health emergencies: experiences from the 2009 H1N1 pandemic vaccination efforts. Biosecur Bioterror. 2010; 8:223-231. 20825333 10.1089/bsp.2010.0036

[36] Hentze I. COVID-19: occupational licensing during public emergencies. www.ncsl.org Web site. https://www.ncsl.org/research/labor-and-employment/covid-19-occupational-licensing-in-public-emergencies.aspx. Accessed July 30, 2020.

[37] Azar AM. Waiver or modification of requirements under section 1135 of the social security act. PHE.gov Web site. https://www.phe.gov/emergency/news/healthactions/section1135/Pages/covid19-13March20.aspx. Accessed February 4, 2021.

[38] NYSED. COVID-19 pandemic and professional practice: Important information for licensees impacted by COVID-19. NYSED.gov Web site. http://www.op.nysed.gov/COVID-19_EO.html#. Accessed February 4, 2021.

[39] Lalani T, Lee TK, Laing ED, et al. SARS-CoV-2 infections and serologic responses among military personnel deployed on the USNS COMFORT to New York City during the COVID-19 pandemic. Open Forum Infect Dis. 2021;8(2):ofaa654. doi: 10.1093/ofid/ofaa654 33553482 PMC7856331

[40] Bellware K, Hawkins D, Knowles H, et al. Coronavirus death toll in U.S. increases as hospitals in hot-spot states are overwhelmed. The Washington Post. July 10, 2020. https://www.washingtonpost.com/nation/2020/07/10/coronavirus-live-updates-us/. Accessed August 20, 2020.

[41] Goff E. Intermountain welcomes ICU nurses from NY to Utah as part of collaborative COVID-19 partnership between two of the nation’s premier health systems. Intermountain Healthcare Web site. https://intermountainhealthcare.org/news/2020/08/intermountain-welcomes-icu-nurses-from-ny-as-part-of-covid-partnership/. Accessed August 10, 2020.

[42] Bowman T. U.S. military is sending medical staff to COVID-19 hotspots. NPR Web site. https://www.npr.org/sections/coronavirus-live-updates/2020/07/13/890553905/u-s-military-is-sending-medical-staff-to-covid-19-hotspots. Accessed August 10, 2020.

[43] Czeisler MÉ, Lane RI, Petrosky E. Mental health, substance use, and suicidal ideation during the COVID-19 pandemic — United States, June 24–30, 2020. MMWR Morb Mortal Wkly Rep. 2020;69(32):1049-1057. 32790653 10.15585/mmwr.mm6932a1 PMC7440121

[44] Shives LR. Basic Concepts of Psychiatric-Mental Health Nursing. 6th ed. Philadelphia: Lippincott Williams & Wilkins; 2005.

[45] Cohen S, Alper CM, Doyle WJ, et al. Positive emotional style predicts resistance to illness after experimental exposure to rhinovirus or influenza a virus. Psychosom Med. 2006;68(6):809-815. doi: 10.1097/01.psy.0000245867.92364.3c 17101814

[46] Falk G, Carter JA, Nicchitta IA, et al. Unemployment rates during the COVID-19 pandemic: Federation of American Scientists. January 21, 2021. https://fas.org/sgp/crs/misc/R46554.pdf. Accessed on June 28, 2021.

[47] Russell D, Smith Hopkins J. Amid coronavirus, calls and texts to mental-health hotlines are surging. Columbia Journalism Investigations. April 2, 2020. https://publicintegrity.org/health/coronavirus-and-inequality/coronavirus-calls-texts-mental-health-hotlines-are-surging/. Accessed August 21, 2020.

[48] Mamelund SE. Effects of the Spanish influenza pandemic of 1918-19 on later life mortality of Norwegian cohorts born about 1900, Memorandum, No. 2003, 29. University of Oslo, Department of Economics, Oslo. 2003. https://www.econstor.eu/bitstream/10419/63083/1/369885325.pdf. Accessed August 21, 2020.

[49] Jeong H, Yim HW, Song YJ, et al. Mental health status of people isolated due to Middle East respiratory syndrome. Epidemiol Health. 2016;38:e2016048. doi: 10.4178/epih.e2016048 28196409 PMC5177805

[50] Pfefferbaum B, Schonfeld D, Flynn BW, et al. The H1N1 crisis: a case study of the integration of mental and behavioral health in public health crises. Disaster Med Public Health Prep. 2012;6(1):67-71. doi: 10.1001/dmp.2012.2 22490939

[51] Rose J. As an ER doctor, I fear our era’s defining symbol will be the refrigerator truck. The Washington Post Web site. https://www.washingtonpost.com/outlook/2020/04/11/refrigerated-truck-morgue-coronavirus/. Accessed August 23, 2020.

[52] Maguire B, O’Neill B, Gerard D, et al. Occupational fatalities among EMS clinicians and firefighters in the New York City Fire Department; January to August 2020. Journal of Emergency Medical Services. https://www.jems.com/coronavirus/occupational-fatalities-among-ems-clinicians-and-firefighters/. Accessed February 8, 2021.

[53] American College of Emergency Physicians. Dr. Lorna Breen health care provider protection act introduced in senate. Web site. https://www.acep.org/corona/COVID-19-alert/covid-19-articles/dr.-lorna-breen-health-care-provider-protection-act-introduced-in-senate/. Accessed August 20, 2020.

[54] Jun J, Tucker S, Melnyk BM. Clinician mental health and well-being during global healthcare crises: evidence learned from prior epidemics for COVID-19 pandemic. Worldviews Evid Based Nurs. 2020;17(3):182-184. doi: 10.1111/wvn.12439 32246793

[55] Chong MY, Wang WC, Hsieh WC, et al. Psychological impact of severe acute respiratory syndrome on health workers in a tertiary hospital. Br J Psychiatry. 2004;185:127-133. doi: S0007125000165080 15286063 10.1192/bjp.185.2.127

[56] Grace SL, Hershenfield K, Robertson E, et al. The occupational and psychosocial impact of SARS on academic physicians in three affected hospitals. Psychosomatics. 2005;46(5):385-391. doi: S0033-3182(05)70049-9 16145182 10.1176/appi.psy.46.5.385 PMC7118753

[57] Congress.gov. Dr. Lorna Breen Health Care Provider Protection Act. S4349. 116th Congress. 1st session. (2020). https://www.congress.gov/bill/116th-congress/senate-bill/4349?q=%7B%22search%22%3A%5B%22d%22%5D%7D&s=1&r=3. Accessed April 29, 2021.

[58] Ripp J, Peccoralo L, Charney D. Attending to the emotional well-being of the health care workforce in a New York City health system during the COVID-19 pandemic. Acad Med. 2020;95(8):1136-1139. doi: 10.1097/ACM.0000000000003414 32282344 PMC7176260

[59] American Hospital Association. COVID-19: caring for our health care heroes during COVID-19. https://www.aha.org/system/files/media/file/2020/05/caring-for-health-care-heros-during-covid-19.pdf. Accessed February 4, 2021.

[60] Fleisher L, Sweeney R, Clapp J, et al. Managing anxiety in anesthesiology and intensive care providers during the covid-19 pandemic: an analysis of the psychosocial response of a front-line department. *NEJM Catalyst*. 2020. https://catalyst.nejm.org/doi/full/10.1056/cat.20.0270. Accessed April 29, 2021.

